# Are Neural Network Potentials Trained on Liquid States Transferable to Crystal Nucleation? A Test on Ice Nucleation in the mW Water Model

**DOI:** 10.1021/acs.jpcb.3c00693

**Published:** 2023-04-19

**Authors:** Francesco Guidarelli Mattioli, Francesco Sciortino, John Russo

**Affiliations:** Sapienza University of Rome, Piazzale Aldo Moro 2, 00185 Rome, Italy

## Abstract

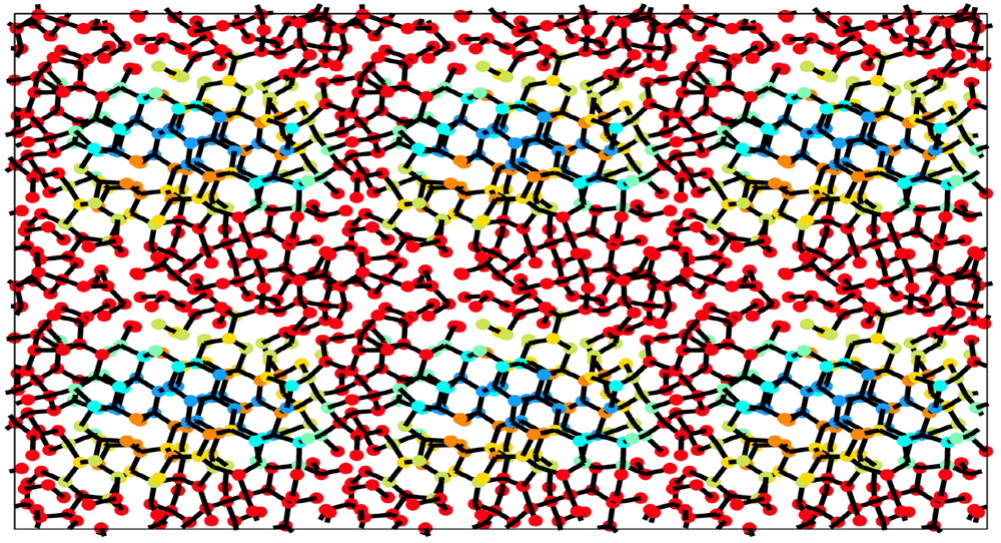

Neural network potentials (NNPs) are increasingly being used to study processes that happen on long time scales. A typical example is crystal nucleation, which rate is controlled by the occurrence of a rare fluctuation, i.e., the appearance of the critical nucleus. Because the properties of this nucleus are far from those of the bulk crystal, it is yet unclear whether NN potentials trained on equilibrium liquid states can accurately describe nucleation processes. So far, nucleation studies on NNPs have been limited to ab initio models whose nucleation properties are unknown, preventing an accurate comparison. Here we train a NN potential on the mW model of water—a classical three-body potential whose nucleation time scale is accessible in standard simulations. We show that a NNP trained only on a small number of liquid state points can reproduce with great accuracy the nucleation rates and free energy barriers of the original model, computed from both spontaneous and biased trajectories, strongly supporting the use of NNPs to study nucleation events.

## Introduction

Neural network potentials (NNPs) are becoming a popular tool to perform classical simulations with the accuracy of ab initio potentials.^1−3^ This is achieved by training a neural network to “learn” the energies (and forces) of atomistic configurations sampled in equilibrium conditions. By mapping a complex first-principles calculation to a classical many-body interatomic potential, NNPs allow for the first time to study processes that would otherwise be computationally prohibitive to simulate with quantum accuracy.^4−6^ Some recent significant applications of NNPs (limited to water) include the computation of the phase diagram^7,8^ and the study of the ice nucleation rate.^9^ The hypothesis of a liquid–liquid phase transition,^10^ confirmed in realistic classical models for water,^11,12^ has also recently received support by studies based on NNPs.^13,14^

In the case of activated events, when the rate of the process is limited by a free energy barrier, the use of NNPs requires careful consideration of the transferability of the potential, i.e., the robustness of the model at conditions other than those used in the fitting process.^15,16^ This problem is easily understood in the context of nucleation, where the free energy barrier corresponds to the formation of a critical nucleus.^17,18^ While NNPs are often trained on equilibrium states, the nuclei that trigger the nucleation process have structural properties that are markedly different from those sampled during the training process. In the case of nucleation we can pinpoint three potential transferability issues. The first one is the presence of a liquid/solid interface, which is usually not included in the training set. The second one is associated with the fact that nuclei often violate the *capillarity* approximation, i.e., their microscopic parameters (like interfacial energy and density) are far from those of the bulk crystal.^19−23^ The third issue occurs when the state point of interest is outside the training region. This last problem cannot be avoided when the state point is close to the kinetic spinodal limit, i.e., when the free energy barrier is small enough that the nucleation time is comparable to the supercooled liquid relaxation time, which makes the preparation of metastable configurations difficult.^24−26^

Assessing transferability issues on nucleation studies is difficult when the original model is based on first-principles calculations. In these cases, computational costs prevent the study of the model for times long enough to observe spontaneous nucleation, and one must then rely on agreement with experiment as a measure of the NNP accuracy.^9^ To our knowledge, a direct comparison between the nucleation properties of a reference model and its NN representation is still missing. Here we will fill this gap by performing a test with the mW model of water^27^—a one-site classical potential that has found widespread adoption to study water’s anomalies^28−31^ and crystallization phenomena.^19,32−34^ Several NNPs have been developed for water, starting from both density functional calculations^7,9,13,35−39^ and classical models with multibody interactions,^8,40^ such as the very accurate MBpol model.^41−43^

The distinct advantage of the mW model is its simplicity and the fact that spontaneous homogeneous nucleation can be directly observed in simulations. By training a NNP on liquid configurations of the mW model, our aim is to compare the nucleation properties, both with direct nucleation trajectories (i.e., when the nucleation barrier is comparable to the thermal energy *k*_B_*T*) and from biased-sampling trajectories (when the nucleation barrier is large compared to *k*_B_*T*). We tackle conditions where all three transferability issues mentioned above are relevant. More precisely, we will show that the NNP representation successfully reproduced the nucleation behavior of the mW model despite being trained only on liquid configurations, without information on the bulk crystal and its interface, and also at thermodynamic conditions outside those used in the training stage. Our test case thus provides a positive outlook on the transferability of NNPs trained on equilibrium configurations to capture both the static and dynamic feautures of crystal nucleation.

The paper is organized as follows. In the Methods section we introduce the neural network potential and demonstrate its accuracy in representing structural and thermodynamic quantities at equilibrium. In the Results and Discussion section we consider the nucleation properties of two state points: one where nucleation occurs spontaneously within the simulation time of both models, and the other one where biased simulations are required to compute the free energy barrier. We end with the Conclusions section, where we provide an outlook on future work.

## Methods

### NNP Training

We use the NNP introduced in ref (44), which is built on a set of atomic fingerprints (AFs) derived from two- and three-body contributions that probe distances and local orientational order, respectively. One of the advantages of this NN implementation is that AFs depend on a small set of tunable parameters that are trained together with the neural network weights, which simplifies the selection of the best atomic fingerprints.

As a first step, we sample mW configurations from NVT simulations (timestep Δ*t* = 4 fs) in three different state points (the same ones as in ref (44)): (i) ρ_1_ = 0.92 g cm^–3^, *T*_1_ = 221.1 K; (ii) ρ_2_ = 0.92 g cm^–3^, *T*_2_ = 270.9 K; (iii) ρ_3_ = 1.15 g cm^–3^, *T*_2_ = 270.9 K. This choice of state points is aimed at improving the agreement with the low temperature–low density region of the phase diagram. Importantly, all configurations come from either stable or metastable liquid state configurations, with the point at ρ_2_ = 0.92 g cm^–3^, *T*_2_ = 270.9 K being close to the limit of stability (respect to cavitation) of the liquid state. The final data set includes 70 configurations of *N* = 1000 water molecules per state points. The data set is then randomly split in train (80% of the configurations) and test (20% of the configurations) data sets. The input of the neural network is composed of 5 two-body AFs and 5 three-body AFs, as this choice has been demonstrated to optimize accuracy and computational cost for the mW model.^44^ The hyperparameters of the NN model are the same as in ref (44), also employing the same learning rate schedule (i.e., the warm restart procedure).

**Figure 1:**
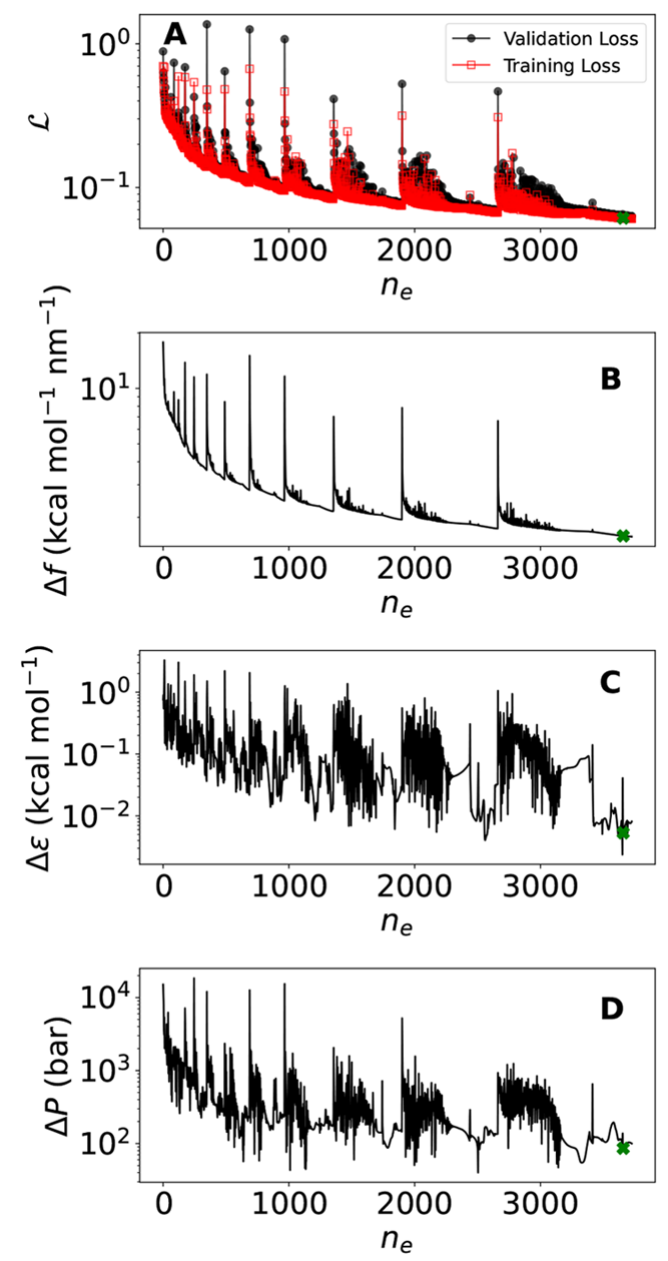
Model convergence properties. (A) Training and validation loss (see  in ref (44)) evolution during the training procedure, reported as a function of the number of epoch *n*_e_ (an epoch is defined as a complete evaluation of the training data set). Root-mean-square (RMS) error (B) of the total potential energy per particle, (C) of the force Cartesian components, and of (D) of the virial pressure during the training evaluated in the test data set. The green cross shows the location of the selected model.

We run 4000 epochs, training on energies and forces and we select the best model by monitoring the error on forces, energies and virial in the test set. In Figure 1A we plot the loss function of the model (defined in ref (44)) during the minimization procedure for both the validation (circles) and training (squares) sets. The observed spikes are due to the warm restart minimization procedure, which increases with a logarithmic spacing the learning rate to facilitate escape from local minima of the loss function. In panels B, C, and D we plot the average errors (defined as the root-mean-square difference between the values of the mW and the NNP model) on forces, energies, and virial pressure, respectively. We recall that the errors in the forces (Figure 1B) and the energy (Figure 1C) enter the definition of the loss function (Figure 1A). We also show the virial pressure error in Figure 1D, as it is an important thermodynamic parameter upon which nucleation is very sensitive. While the error in the forces decreases consistently with the number of epochs, the energy and pressure errors show a more rapid decay followed by strong fluctuations. In choosing the best model, it is thus important to set a criteria that not only takes into account the value of the loss function but importantly also of the energy and pressure errors. Our criteria are the following: among the models explored in the last steps of the training process (the ones with the lowest force errors), we select the one that is a local minima of both the energy and pressure errors. The final model results in an error on the energy of Δϵ ≃ 0.0053 kcal mol^–1^(0.23 meV), on the force of Δ*f* ≃ 1.58 kcal mol^–1^ nm^–1^ (6.88 meV Å^–1^), and on virial pressure of Δ*P* ≃ 86 bar.

**Figure 2:**
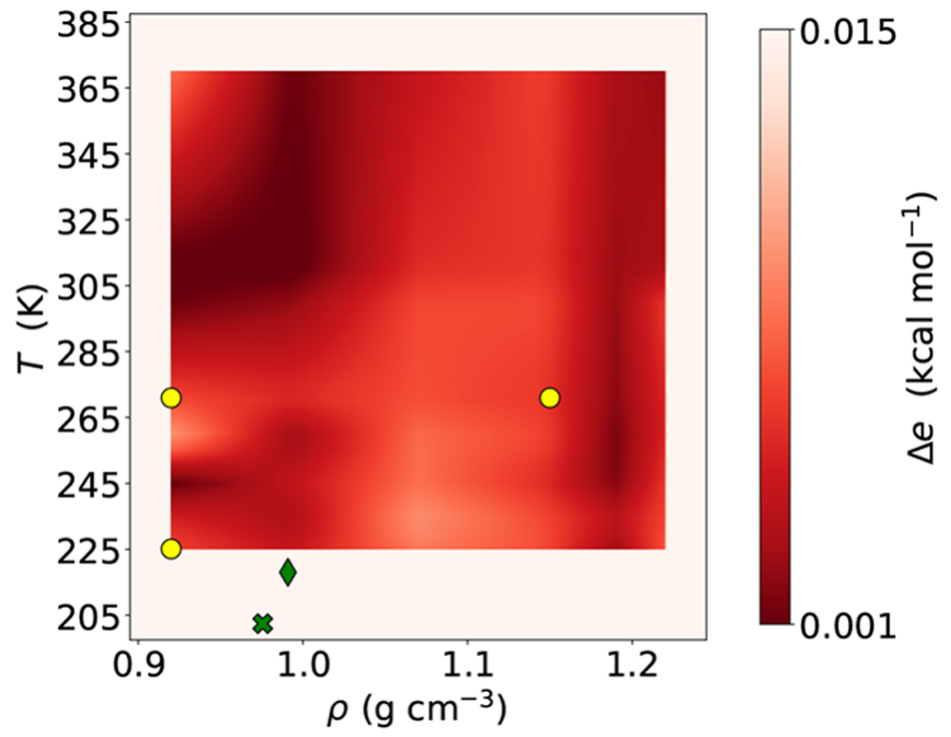
Mean absolute error in the total energy per particle (Δ*e*) between the mW and the NNP model for different temperatures and densities. The continuous map has been interpolated on a grid of 48 state points made of six densities, ranging from 0.92 to 1.22 g cm^–3^, and eight temperatures from 221.1 to 373.7 K. The largest error on this map is of 0.010 kcal mol^–1^. The NNP model can reproduce the mW total energy with a good agreement in a wide region of densities and temperatures. Yellow circles represent the state points used for building the NNP model, while the green diamond and cross are the state points where bias and spontaneous nucleation properties are analyzed in this study, respectively.

Since the extensive nature of the total potential energy, all errors on energies are reported as normalized by the number of particles in the systems. Figure 2 plots the energy error Δ*e* = |*e*_mW_ – *e*_NNP_|, where *e* = *E*/*N* is the total energy per particle, in an extensive region of the *T*–ρ phase diagram, obtained by comparing the average total energy obtained from molecular dynamics simulations in NVT ensemble of the mW model (*e*_mW_) and the NNP model (*e*_NNP_) for each state point. The yellow circles represent the three state points at which configurations were used for the training of the network. The figure demonstrates that the energy error is below Δ*e* ≲ 0.01 kcal mol^–1^ over a region of the phase diagram spanning liquid state configurations from the cavitation limit (ρ ≲ 0.95 g cm^–3^) to extreme pressure conditions (ρ ≳ 1.2 g cm^–3^), also extending to deeply supercooled states. The two green symbols in Figure 2 correspond to the two state points where nucleation properties will be analyzed in the next section: the first one (cross symbol) in which spontaneous nucleation is observed and the second one (diamond symbol) in which biasing techniques are needed to evaluate the nucleation rate. Both state points are at temperatures below the training region to test the transferability of the NNP.

### Nucleation Simulations

Nucleation simulations are run in the NPT ensemble using the Langevin-piston barostat^45^ for both the mW potential^27^ and the NNP model previously described. The simulation size for each model is *N* = 1000 water molecules. The environment around each particle is classified as crystalline with the *Q*_12_ bond-orientational order parameter, and the size of the crystalline nuclei is computed with a cluster algorithm that connects nearest neighbors that are part of the same local crystalline environment. The size of the largest crystalline cluster *n* is then selected as the order parameter. For a detailed description on the use of bond-orientational order parameters to study water nucleation see ref (46).

For the umbrella sampling simulations we make use of the CNT-US scheme,^47^ which allows to sample the whole nucleation barrier within a single simulation by adding a potential term to the Hamiltonian with the functional form predicted by classical nucleation theory (see the Supporting Information of ref (47) for a detailed description). Ideally, if the classical nucleation theory prediction would be exact, then the barrier to nucleation would be completely suppressed, allowing a proper sampling of otherwise rarely explored configurations. To adapt the CNT-US scheme, which is a Monte Carlo scheme, to the molecular dynamics implementation of the NNP, we use the hybrid Monte Carlo (HMC) method,^48^ where NVE molecular dynamics trajectories are accepted or rejected based on the Metropolis acceptance criteria for the thermostat, barostat, and the bias potential. Briefly, we perform a MD trajectory in the NVE ensemble for 0.02 ps, which is then accepted with probability
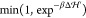
1where , *K* is the total kinetic energy, *U* is the total potential energy, and η(*n*) is the bias potential defined as
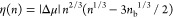
2where Δμ = 0.57*k*_B_*T* is the known chemical potential difference between the bulk crystal and the liquid at *T* = 218 K and ambient pressure,^47^ while *n*_b_, the only adjustable parameter of the bias, was fixed to 90 after preliminary test. Velocities are extracted from a Maxwell–Boltzmann distribution after every HMC move. To equilibrate the volume, isobaric moves are performed with a probability of 0.01 after each HMC move.

## Results and Discussion

### *T* = 202.4 K: Spontaneous Nucleation

To observe spontaneous nucleation events from liquid to crystalline particles, we choose a the state point at *T* = 202.4 K (*T* = 0.065 in mW internal units) and at ambient pressure where the stable phases are the diamond cubic and diamond hexagonal crystals.^22^

**Figure 3:**
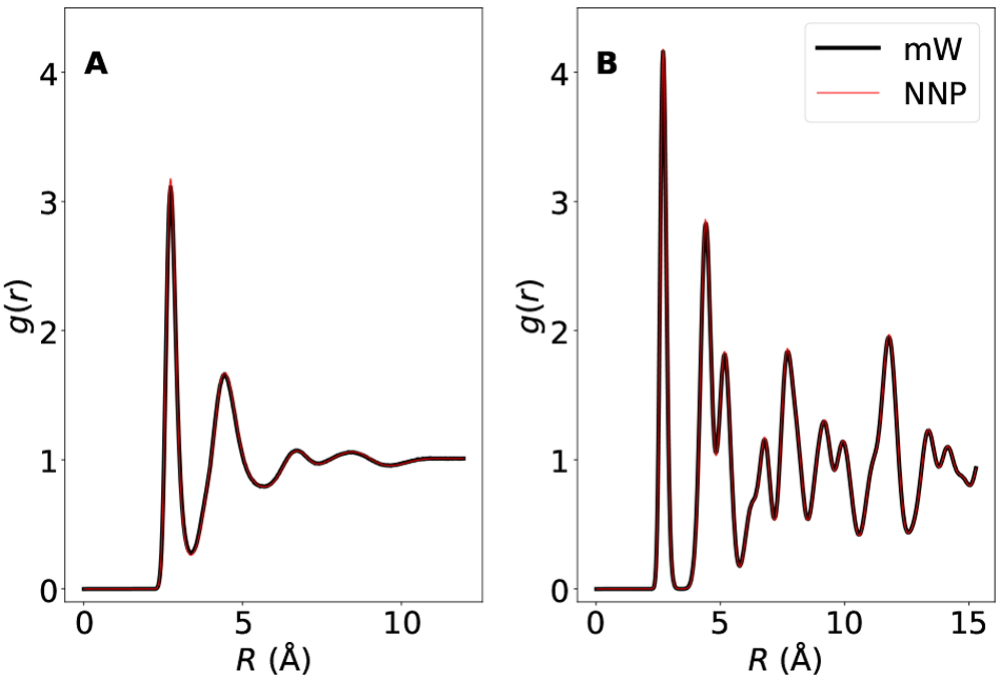
Comparison between the mW radial distribution function *g*(*r*) and the NNP *g*(*r*) at *T* = 202.4 K and ambient pressure for (A) supercooled liquid configurations and for (B) the bulk cubic diamond crystal. The supercooled liquid *g*(*r*) is computed by using only trajectories before the formation of the critical nucleus. The good agreement between the mW and NNP *g*(*r*) shows that the NNP model can represent both the liquid and the solid structures at this supercooled state point.

We first test the ability of the NNP to reproduce structural properties at this state point, which is at a temperature considerably lower than the ones used for the training of the potential. Figure 3 compares the radial distribution functions in the two models at supercooled liquid conditions (panel A) and for the diamond cubic phase (panel B), both at *T* = 202.4 K and at ambient pressure. Not only does the NNP reproduce accurately all pair correlation features of the mW liquid (which are typical of tetrahedral liquids), but it also offers (panel B) a detailed representation of the bulk crystal structure, despite the absence of crystal configurations in the training set. A similar quality has also been observed for the hexagonal diamond lattice (not shown). Figure 3 thus shows that the NNP model can extrapolate equilibrium structural information of both phases. Next we turn to the investigation of nonequilibrium states accessed during nucleation simulations, where the presence of the interface and the activated nature of the transition make the transferibility of the potential much more challenging.

**Figure 4:**
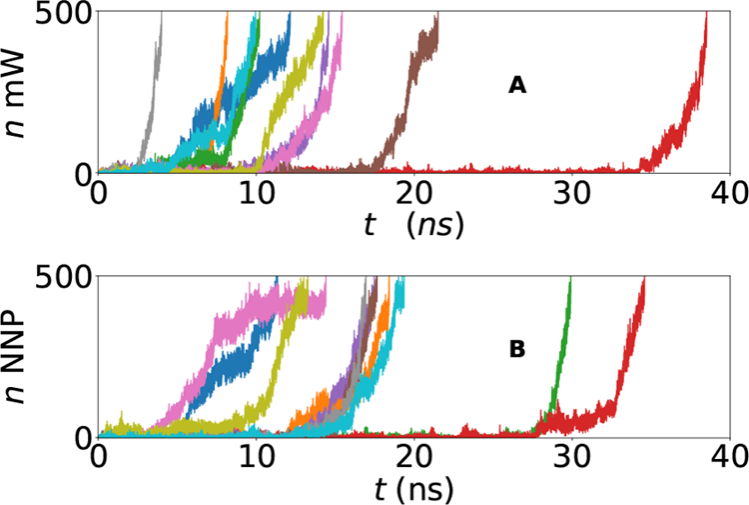
Time evolution of the size of the largest crystalline nucleus (*n*) for a sample of 10 (out of 100) different simulations for both (A) the mW potential and (B) the NNP model.

We run 100 MD simulations in the NPT ensamble at the same pressure, temperature, system size, and integration step (Δ*t* = 4 fs) for both the mW potential and the NNP model. Figure 4 compares the time evolution of the order parameter *n*, defined as the size of the largest crystalline cluster in the simulation box, for a random selection of 10 trajectories for each model. The simulations are interrupted once the largest crystal comprises 50% of the total system. While it is known that under these conditions the mW potential nucleates spontaneously^22^ (as confirmed by the data reported in Figure 4A) in Figure 4B we see that also the NNP model can nucleate in a similar way. Not only is the quality of the nucleation trajectories similar between mW and NNP, but also the nucleation times are comparable. This first qualitative finding proves that a NNP model can spontaneously nucleate—a finding that to date has been reported only for simulations started with a crystalline seed,^9^ with enhanced sampling techniques,^49^ or with linear machine-learning models.^50,51^

**Figure 5:**
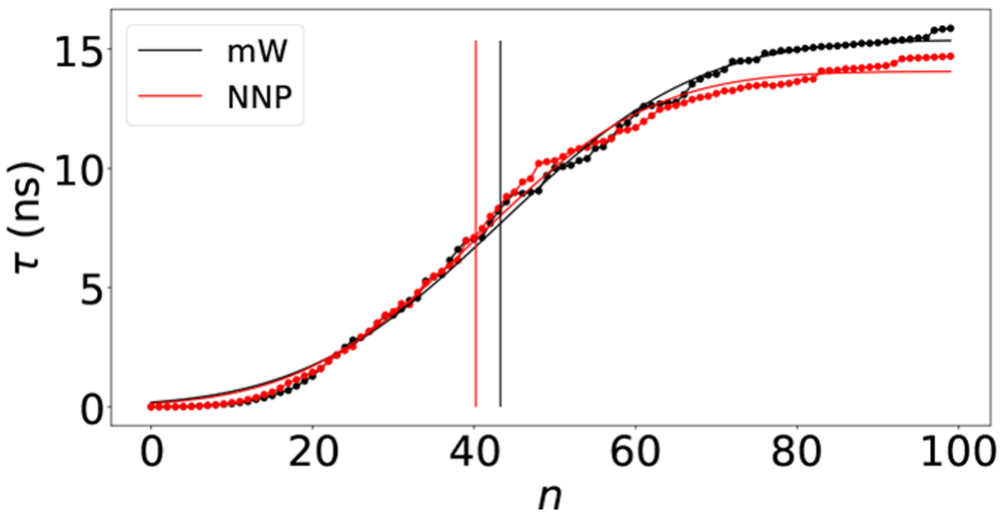
Mean first passage time τ of the mW (black curve) and NNP model (red curve) at *T* = 202.4 K and at ambient pressure, computed from 100 independent simulations trajectories in the isothermal–isobaric ensemble. Points correspond to raw data while lines correspond to the fit of data with functional form defined in eq 3. According to the fit, vertical lines indicate the size of the critical nucleus that is *n*_c_ = 43 for mW potential and *n*_c_ = 40 for the NNP model, while the nucleation rates are *J* = 2.13 × 10^–6^ ns^–1^ Å^–3^ for mW and *J* = 2.32 × 10^–6^ ns^–1^ Å^–3^ for NNP.

To quantify our results, we compute the mean first passage time τ of the largest nucleus in the system for all 100 trajectories and fit it with the theoretical expression of Wedekind and Reguera^52^
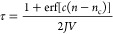
3where *J* is the nucleation rate, *n*_c_ is the critical nucleus size, erf is the error function, and  is proportional to the curvature (i.e., the second derivative) of the free-energy barrier Δ*G* at the top of the barrier. This approach has been used quite often in the past, and it has been demonstrated that it provides an accurate (within *k*_B_*T*) estimate of the barrier height.^53,54^ We show the results in Figure 5, where the symbols are obtained from simulations, and the continuous lines are the fits for *n*_max_ < 100 for both the mW and NNP models. The figure demonstrates that the NNP reproduces with excellent agreement the nucleation dynamics of the mW model at this state point. From the fits we can extract the critical nucleus size from the flex point of the curves (the vertical lines in Figure 5), and we obtain *n*_c_ = 43 for mW and *n*_c_ = 40. The extracted nucleation rates are *J* = 2.1 × 10^–6^ ns^–1^ Å^–3^ for mW and *J* = 2.3 × 10^–6^ ns^–1^ Å^–3^ for NNP.

**Figure 6:**
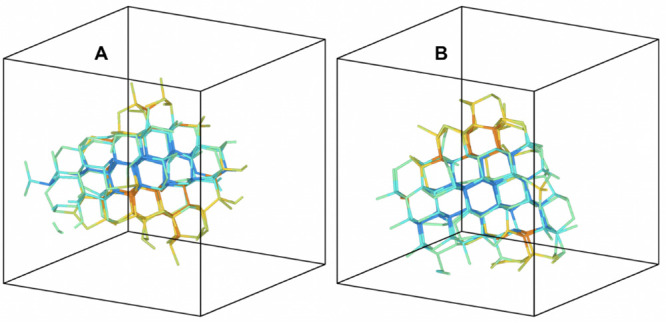
Snapshots of a nucleation event observed in MD simulations based on the NNP model (A) and on the mW model (B). Only bonds between molecules that belong to the largest nucleus size (approximately *n* = 170 in both snapshots) are plotted. The color of the bond indicates the detected crystalline phase: blue for the cubic diamond and orange for the hexagonal diamond. Molecules in liquid environments are not represented. The snapshots were prepared with the Ovito program.^55^

Looking at snapshots of the formed crystalline nuclei, we observe a similar structure on average between the mW and NNP models. Two example snapshots are shown in Figure 6. We observe nucleation of both cubic and diamond hexagonal polymorphs, in agreement with previous results.^22^

### *T* = 218 K: Biased Nucleation

Next, we study the nucleation behavior of the mW and NNP models at *T* = 218 K (*T* = 0.07 in mW internal units) and at ambient pressure. Despite being deeply supercooled, at this state point nucleation does not occur spontaneously within the simulation time in the mW model. The absence of spontaneous nucleation signals a free energy barrier which is too high for thermal fluctuation to overcome within typical simulation times. In order to study the nucleation behavior at these conditions, we add to the potential an external bias that reduces the free-energy cost of forming a nucleus.

**Figure 7:**
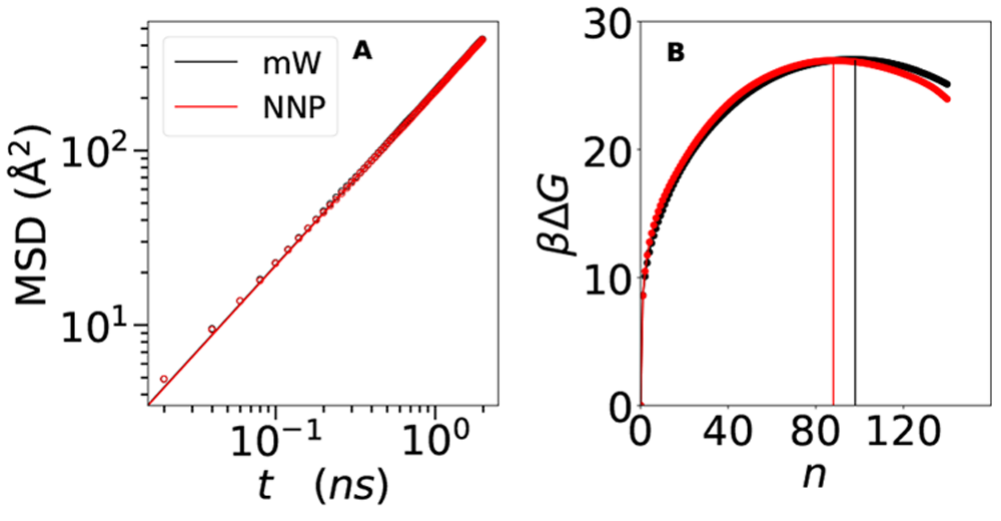
Simulations at *T* = 218 K and at ambient pressure. (A) Mean-square displacement (MSD) of the mW model (black symbols) and NNP model (red symbols), together with the fit ⟨Δ*r*^2^⟩ = 6*Dt* (lines). (B) Nucleation barriers for mW (black symbols) and NNP (red symbols) models computed from CNT-US biased simulations.

According to classical nucleation theory, the nucleation rate has two distinct contributions: a dynamic and a thermodynamic one. The dynamic part of the nucleation rate is controlled by the long-time diffusion coefficient of the melt.^56^ The diffusion coefficient is extracted from the mean-square displacement that we plot in Figure 7A at *T* = 218 K and at ambient pressure. The figure shows that both models have virtually the same diffusion coefficient (*D* = 36 Å^2^ ns^–1^). The NNP model offers an excellent representation of the dynamical properties of the mW potential, despite the fact that no dynamical quantity was included in the training set.

The thermodynamic contribution to the nucleation rate is determined by the height of the free energy barrier, i.e., the free energy cost of formation of the critical nucleus. To compute this quantity, we run CNT-US simulations, as discussed in the Methods section. The Gibbs free energy is connected to the probability distribution of the size of the largest cluster, βΔ*G* = −log *P*(*n*_max_). The CNT-US method allows to run simulations in a flattened βΔ*G* landscape and to recover the original histogram distribution *P*(*n*_max_) by subtracting the effect of the external bias. The resulting unbiased Gibbs free energy barriers for the state point *T* = 218 K and at ambient pressure are plotted in Figure 7B. Again, the agreement between the mW and NNP models is excellent: the free-energy barrier is βΔ*G* ≃ 27 for both models, with only a small shift of the critical size: *n*_c_ ≃ 98 for the mW model and *n*_c_ ≃ 88 for the NNP model. This small size difference can be ascribed to a possible discrepancy between the two models in the description of interfacial properties, but we stress that the nucleation rate is primarily controlled by the height of the barrier and not by the critical size.

Overall, we can conclude that also biased simulations at these conditions show that the NNP model has successfully captured nucleation properties, despite being tested at temperatures below those of its training set and despite not having any information on the dynamics and interfacial states of the system.

## Conclusions

In this paper we have directly tested for the first time whether a neural network potential (NNP) representation of a simple potential (the mW model for water) can faithfully reproduce the nucleation behavior of the original model.

What makes this test interesting is the fact that the NNP could in principle suffer from transferibility problems, three of which are directly tested in our case. First, the NNP is trained only on disordered liquid configurations and does not “learn” from configurations with interfaces. Second, the NNP has to represent out-of-equilibrium states where the capillarity approximation is violated, i.e., the fact that nuclei will form at structural conditions very different from those found in bulk systems. Third, we tested our NNP outside the thermodynamic conditions on which it was trained, making predictions of deeply supercooled states that are dynamically and structurally different than the trained ones.

We considered two different state points at ambient pressure. The first one, at *T* ≃ 202 K, was chosen because at the same conditions the mW model nucleates spontaneously in simulations. This allowed to first test whether the same behavior was also observed in the NNP model and from there extract the nucleation properties (nucleation rates and critical nucleus size) from the analysis of mean first passage times. The second state point, at *T* = 218 K, instead was analyzed with biased simulations, from which the full free energy barrier was computed.

In all cases we find a remarkable agreement in the nucleation behavior of the NNP model compared to the original mW model, proving that these potentials are indeed suitable to study nucleation behavior. Moreover, being trained only on liquid configurations, our network shows that properties of both the bulk crystals and of their interfaces with the melt can be extracted from the local environments that the system explores in its liquid phase.

Although the mW potential represents a valid target model to test in a clear way the NN transferability, the NN potential does not lead to a more computationally efficient model. Indeed, while both mW and NN potentials are based on distances and triplets, the mW is a linear (additive) potential and differently the NN potential is nonlinear (and nonadditive). The computational cost of the NN potential is increased due to the evaluation of the AFs and of the neural network layers both in forward and backward passes to respectively compute energies and forces. Using our own custom code, we find a slowing down less than 1 order of magnitude. In general, NN potentials represent a valid and nowadays standard tool to simulate more efficiently polarizable potentials^8^ as well as atom systems with high ab initio accuracy.^57,58^

Before concluding, we note that the observed transferability from liquid to crystal phases does not imply that a comparable success would be observed for modeling coexistence between liquid and gas phases, where the local geometries at the interface are more different than at the liquid-crystal one and electrostatic long-range interactions are not screened. The mW potential, while enabling a one-to-one comparison between a reference model and its NN representation, does not include any long-range contribution, and thus we cannot guarantee in principle that such agreement will be found also in models including charges and point polarizabilities. Indeed, NN potentials, being trained on condensed-phase configurations, can account for long-range correlations implicitly by including them in their shorter-range description (which in the case of liquids with a high dielectric constant can mimic a reaction-field type of contribution) or explicitly by adding suitable electrostatic effects via Ewald sum contributions.^59,60^ Recent studies,^8^ for the gas–liquid coexistence, have revealed an intrinsic difficulty in simultaneously modeling liquid bulk quantities and gas–liquid interfacial quantities with standard NN potential without long-range corrections. Clearly more work is needed to definitively assess the role of long-range electrostatic interactions in water simulations in order to confirm whether the “all information in liquids” hypothesis^61^ holds, at least for the crystal phases.

In future work we plan to study whether the same agreement between mW and NNP holds at lower supersaturation, which will require large-scale simulations and the adoption of new methods such as the seeding technique.^62−64^ Studies similar in spirit to the one reported here will demonstrate if the observed agreement is also found in other atomic or molecular systems and if NNP can properly describe nucleation processes characterized by more complex crystallization pathways.^50,65^

## Data Availability

Data supporting this work are available on Zenodo on DOI 10.5281/zenodo.7804928 while codes are available on github at https://github.com/FGMphys/NN-mW-Nucleation.

## Dedication

We dedicate this article to Pablo Debenedetti on the occasion of his 70th birthday. His seminal studies have shaped (and will continue to do so for many decades) research on supercooled liquids (and water in particular).
